# Spectrum of Esophageal Motility Disorders on High-Resolution Manometry in Patients With Non-obstructive Dysphagia

**DOI:** 10.7759/cureus.89478

**Published:** 2025-08-06

**Authors:** Devendra S Kushwaha, Arka Banerjee

**Affiliations:** 1 Gastroenterology, School of Digestive and Liver Diseases, Institute of Post-Graduate Medical Education and Research and Seth Sukhlal Karnani Memorial Hospital, Kolkata, IND

**Keywords:** achalasia cardia, eastern india, esophageal motility disorders, high-resolution manometry, timed barium swallow

## Abstract

Background and objectives

Esophageal motility disorders (EMDs) are a major cause of non‑obstructive dysphagia. However, regional data from eastern India are limited. This study aims to describe the spectrum of EMDs in patients with non‑obstructive dysphagia using high‑resolution manometry (HRM) at a tertiary care center in eastern India, and to compare clinical symptoms, and endoscopic and barium findings in patients with achalasia versus non‑achalasia.

Methods

This cross‑sectional observational study was conducted between August 2023 and March 2025 at a tertiary care center in eastern India. Adult patients with dysphagia underwent HRM after ruling out an obstructive lesion on upper gastrointestinal endoscopy (UGIE). Symptom profile and endoscopic and barium swallow findings were recorded. Descriptive statistics summarized demographic and clinical features. Continuous variables were expressed as mean ± SD, and categorical variables as frequencies/percentages. Chi‑square test, Fisher's exact test, and Mann‑Whitney test were used for inter‑group comparisons. All analyses were two‑tailed, with p < 0.05 considered statistically significant. Data were entered into Microsoft Excel and analyzed using IBM SPSS Statistics (v31.0.0.0).

Results

A total of 262 patients (mean age 43.6 ± 14.3 years; 57% female) were included. HRM revealed achalasia in 53.9% (Type I: 5.0%, Type II: 46.2%, and Type III: 2.7%), esophagogastric junction outflow obstruction (EGJOO) in 9.1%, ineffective esophageal motility (IEM) in 8.4%, absent contractility in 8.4%, fragmented peristalsis in 1.9%, and normal motility in 17.9%. Dysphagia to both solids and liquids and weight loss were reported by 76.7% and 78.6% of patients, respectively. Gastroesophageal junction (GEJ) resistance was seen on endoscopy in 62.2% and significant barium retention in 67.2%. Univariate analysis showed achalasia patients had significantly higher Eckardt scores (ESs), and more frequent abnormal endoscopic and barium findings. Dysphagia, regurgitation, and weight loss were significantly more common in achalasia (p < 0.001). No significant associations were found for gender, chest pain, or BMI (p > 0.05). Multivariate analysis identified abnormal UGIE findings (including GEJ resistance - with dilated esophagus or retained food) and barium retention at one and five mins on timed barium swallow (TBS) as independent predictors of achalasia.

Conclusion

Achalasia, especially Type II, is the most prevalent motility disorder in patients with non‑obstructive dysphagia in eastern India. HRM is essential for accurate diagnosis and classification of EMD. This study highlights the need for increased HRM accessibility and early referral for HRM based on achalasia predictors in resource‑limited settings.

## Introduction

Esophageal motility disorders (EMDs) represent a heterogeneous group of conditions characterized by abnormalities in esophageal peristalsis or esophagogastric junction (EGJ) function, frequently leading to symptoms such as dysphagia, regurgitation, and chest pain [1]. The diagnostic approach to EMDs has been transformed by high‑resolution manometry (HRM), which offers a detailed assessment of esophageal pressure topography and EGJ relaxation. The Chicago Classification (CC v3.0) has standardized HRM interpretation and categorizes EMDs into disorders of EGJ outflow obstruction, major disorders of peristalsis, minor disorders of peristalsis, and normal motility [2].

Achalasia cardia is the most prevalent and clinically relevant EMD, defined by impaired lower esophageal sphincter (LES) relaxation and absent peristalsis. It is subclassified into three manometric subtypes: Type I (classic), characterized by minimal pressurization; Type II, by panesophageal pressurization and the most prevalent and favorable response to treatment; Type III, by spastic distal contractions and typically poorer therapeutic outcomes. Differentiating among EMD subtypes is essential, as management strategies differ significantly based on the underlying disorder [2,3].

In India, studies from different regions have shown that achalasia remains the dominant diagnosis, especially Type II, followed by esophagogastric junction outflow obstruction (EGJOO) and ineffective esophageal motility (IEM) [1,3-5]. However, limited data are available from eastern India, where diagnostic delays and restricted access to HRM technology remain prevalent barriers to early diagnosis and treatment [4]. Regional studies from Asian countries have reported varied prevalence patterns of EMDs, influenced by diagnostic protocols, referral patterns, and availability of HRM [6,7].

Bansal et al. and El-Takli et al. have consistently demonstrated that endoscopic and barium findings, when interpreted within the clinical context, are valuable in pre-manometric screening [3,8].

Nonetheless, since CC v4.0 is relatively new and not yet universally implemented, this study follows CC v3.0 for consistency with prior regional literature and diagnostic practices [9].

This study aims to describe the spectrum of EMDs in patients with non-obstructive dysphagia using HRM at a tertiary care center in eastern India, and to compare clinical symptoms, and endoscopic and barium findings in patients with achalasia versus non-achalasia.

By contributing regional data, this study intends to bridge the gap in current literature, early referral for HRM and management strategies for patients with non-obstructive dysphagia.

## Materials and methods

This cross-sectional observational study was conducted at the Department of Gastroenterology, Institute of Post-Graduate Medical Education and Research and Seth Sukhlal Karnani Memorial Hospital, Kolkata, from August 2023 to March 2025, following Institutional Ethics Committee approval and written informed consent from all participants.

Adult patients (≥18 years) presenting with dysphagia or referred for dysphagia evaluation were enrolled. Before HRM, all patients underwent upper gastrointestinal endoscopy (UGIE) to rule out structural or mucosal lesions. Patients with a history of corrosive ingestion, prior esophageal surgery, pneumatic dilatation (PD) or per-oral endoscopic myotomy (POEM), pregnancy, refusal to participate, esophageal stricture, or esophageal mass on endoscopy were excluded.

Their demographic details and clinical history, including dysphagia type (solids, liquids, or both), duration, regurgitation, chest pain, and weight loss was recorded. Symptom severity was quantified using the Eckardt score (ES) (0 to 12).

Following overnight fasting, UGIE was performed using a standard endoscope in left lateral decubitus without sedation. Timed barium swallow (TBS) was obtained under fasting conditions. Participants consumed 100-250 mL of low-density barium sulfate within 15 to 20 seconds in a standing position. One spot, four films were obtained at baseline, one, two, and five mins to evaluate esophageal emptying. HRM was performed after overnight fasting with a water-perfused catheter containing 16 circumferential sensors spaced at 2 cm. Following calibration, the catheter was inserted trans-nasally to span from the hypopharynx across the lower esophageal sphincter into the stomach. After 60-second adaptation period and 30-second baseline recording, at least 10 swallows of 5 mL water bolus were recorded to assess esophageal motility and LES metrics in accordance with CC version 3.0.

**Figure 1:**
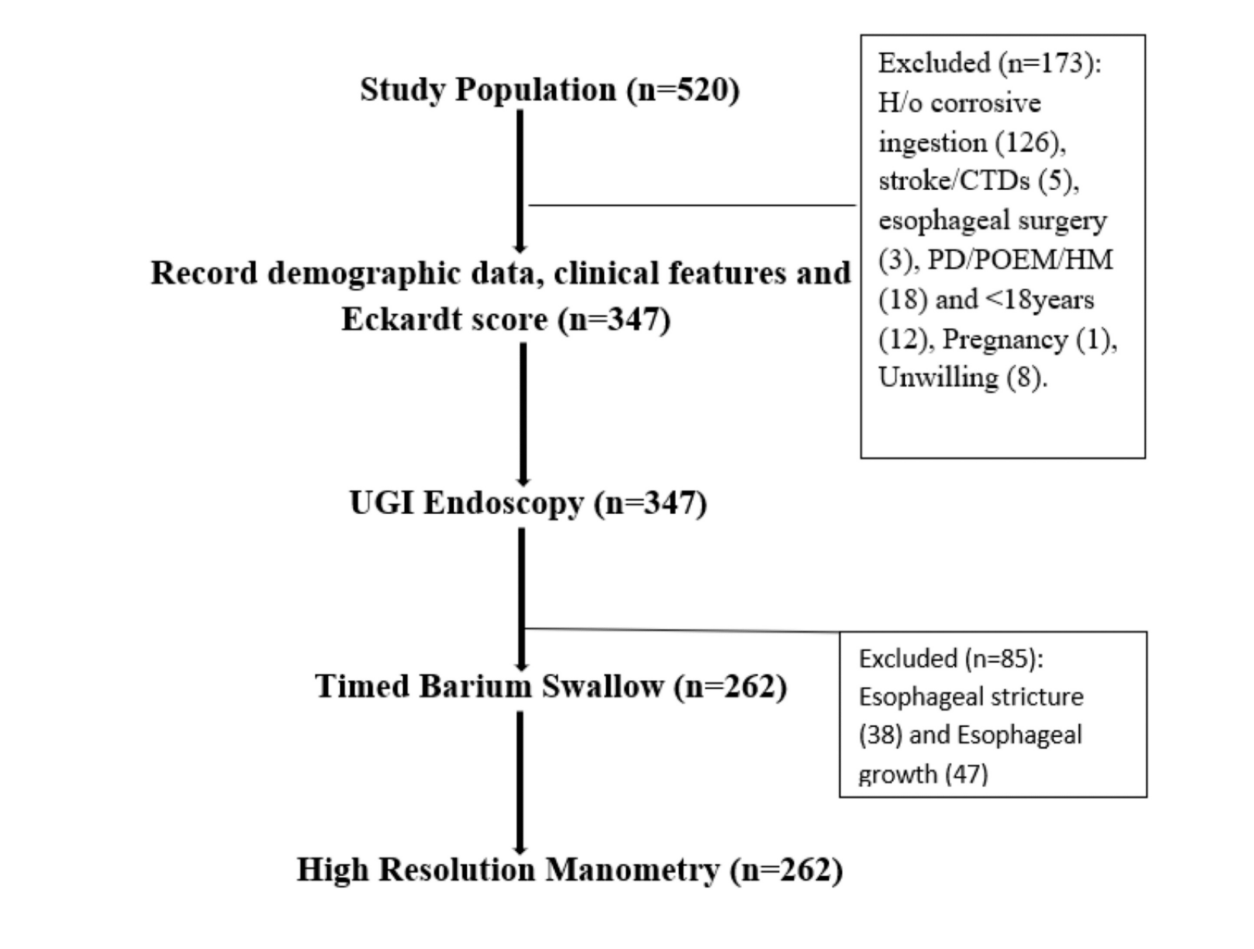
Flow chart CTD: Connective tissue disorder; PD: Pneumatic dilatation; POEM: Per-oral endoscopic myotomy; HM: Heller's myotomy; UGI: Upper gastrointestinal

Data were entered into Microsoft Excel and analyzed using IBM SPSS Statistics (version 31.0.0.0). Descriptive statistics summarized demographic and clinical features. Continuous variables were expressed as mean ± SD and categorical variables as frequencies and percentages. Intergroup comparisons were done by using Chi-square test, Fisher’s exact test for categorical variables and Mann-Whitney U test for continuous variables. All analyses were two-tailed, and p-value less than 0.05 was considered statistically significant (Figure 1).

## Results

In this study, 262/520 patients underwent HRM and were included in the final analysis.

**Figure 2:**
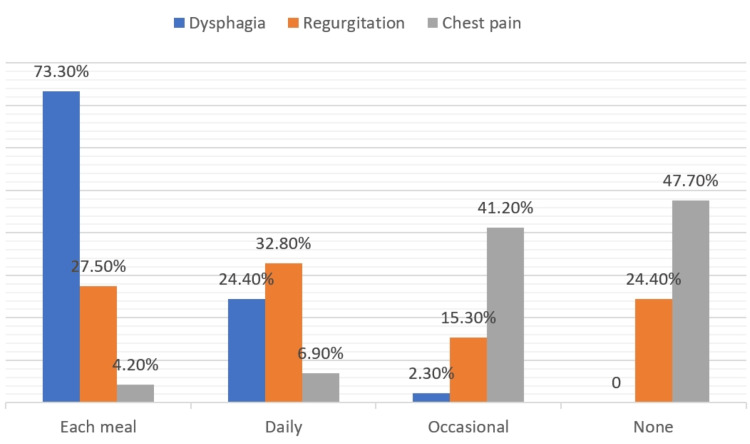
Frequency of symptoms

Most patients were middle-aged (mean: 43.6 ± 14.3 years; range: 18-82 years). Age distribution was slightly right-skewed, with fewer elderly patients. Symptom duration varied broadly (interquartile range (IQR): 12-60 months), indicating that at least 25% of patients had symptoms for over five years before diagnosis. The majority were female (57%, n=149). Mean BMI was 20.4 ± 2.3 kg/m². Most patients experienced dysphagia to both solids and liquids (76.7 %, n=201), typically with each meal (73.3%, n=192). Regurgitation occurred in approximately three-quarters, and chest pain in nearly half. ESs ranged from 3 to 12, with 61.1% (n=161) scoring between 5 and 8 (Table 1, Figure 2). 

**Table 1:** Characteristics of study population

Parameters	Value
Age in years (mean ± SD)	43.6 ± 14.3
BMI in kg/m^2 ^(mean ± SD)	20.4 ± 2.3
Gender (% (n))	
Female	43 (149)
Male	57 (113)
Symptoms (% (n))	
Dysphagia to both solids and liquids	76.7 (201)
Regurgitation	75.6 (198)
Chest pain	52.3 (137)
Weight loss	78.6 (206)

In UGIE, the majority showed gastroesophageal junction (GEJ) resistance with a dilated esophagus and/or residual food. Two-thirds had abnormalities on TBS, with 82% demonstrating significant barium retention at both one and five minutes (Table 2).

**Table 2:** Distribution of endoscopic and barium findings UGIE: Upper-gastrointestinal endoscopy; TBS: Timed barium swallow; GEJ: Gastroesophageal junction

Endoscopic/Barium	Findings	Percentage (n)
UGIE findings	GEJ resistance alone	19.5 (51)
	GEJ resistance with dilated esophagus and/or food residue	42.7 (112)
	Normal study	37.8 (99)
TBS findings	No barium retention in 1 min	32.8 (86)
	Barium retention in 1 min >5 cm	8.8 (23)
	Barium retention in 5 min >2 cm	2.7 (7)
	Barium retention in 1 min >5 cm and in 5 min >2 cm	55.7 (146)

**Figure 3:**
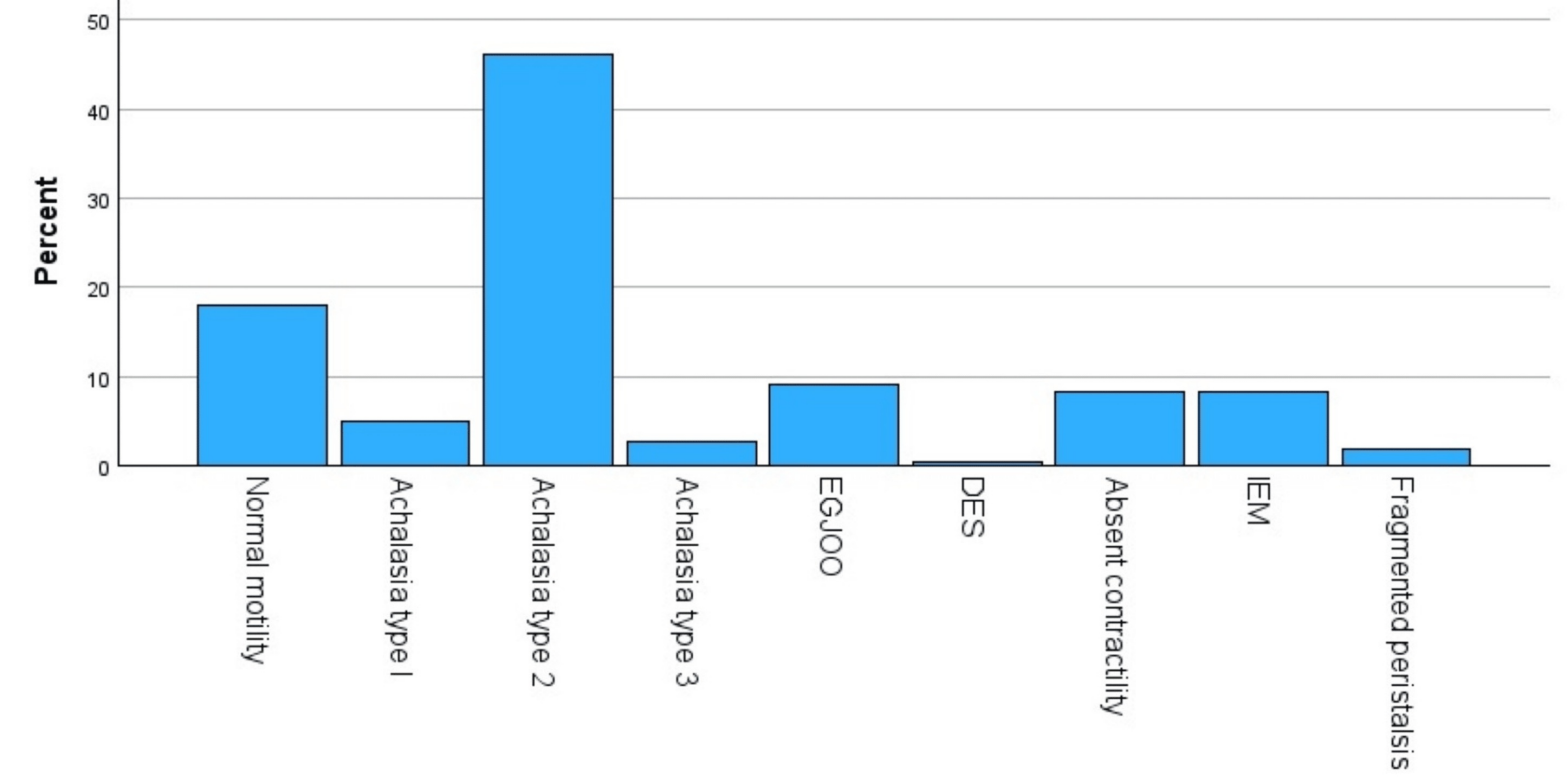
Spectrum of EMDs on HRM EGJOO: Esophagogastric junction outflow obstruction; DES: Distal esophageal spasm; IEM: Ineffective esophageal motility; EMD: Esophageal motility disorder; HRM: High‑resolution manometry

HRM revealed Type II achalasia as the most prevalent disorder (46.2%, 121/262), followed by EGJOO (9.1%, 24/262), absent contractility (8.4%, 22/262), and IEM (8.4%, 22/262). Normal motility was seen in 17.9% (n=47) patients. Stratified by gender, Type II achalasia remained predominant in both males (41.7%, 47/113) and females (49.6%, 74/149). EGJOO occurred in 11.5 % of males versus 7.4 % of females, absent contractility in 7.1% versus 9.4%, and IEM in 9.7% versus 7.4%. Other disorders (Type I/III achalasia, DES, fragmented peristalsis) were rare (<5%), and jackhammer esophagus was absent in all. One patient had borderline distal contractile integral (~8,000 mmHg·s·cm) but normalized on follow up (Table 3, Figure 3).

**Table 3:** Spectrum of EMDs on HRM HRM: High-resolution manometry; EGJOO: Esophagogastric junction outflow obstruction; DES: Distal esophageal spasm; IEM: Ineffective esophageal motility; EMD: Esophageal motility disorder

HRM Diagnosis	Percentage (n)	Gender	
		Male (% (n))	Female (% (n))
Normal motility	17.9 (47)	18.6 (21)	17.4 (26)
Achalasia Type I	5 (13)	4.4 (5)	5.4 (8)
Achalasia Type II	46.2 (121)	41.7 (47)	49.6 (74)
Achalasia Type III	2.7 (7)	3.5 (4)	2 (3)
EGJOO	9.1 (24)	11.5 (13)	7.4 (11)
DES	0.4 (1)	0 (0)	0.7 (1)
Absent contractility	8.4 (22)	7.1 (8)	9.4 (14)
Jackhammer esophagus	0 (0)	0(0)	0(0)
IEM	8.4 (22)	9.7 (11)	7.4 (11)
Fragmented peristalsis	1.9 (5)	3.5 (4)	0.7 (1)
Total	100 (262)	100 (113)	100 (149)

Univariate analysis (Chi-square, Fisher’s exact, Mann-Whitney U tests) showed that achalasia patients had significantly higher ESs and more frequent abnormal endoscopic and TBS findings (p <0.001). Dysphagia, regurgitation, and weight loss were also significantly more common and severe among achalasia patients. No significant associations were observed for gender, chest pain, or BMI (Tables 4, 5).

**Table 4:** Univariate analysis between patients with achalasia versus non-achalasia *: Significant (p value <0.05); **: Highly significant (p value <0.01) UGIE: Upper-gastrointestinal endoscopy; TBS: Timed barium swallow; ES: Eckardt score

Variables	Test Used	P-value
Gender	Fisher's exact	0.26
Hypothyroidism	Fisher's exact	0.018^*^
Dysphagia severity	Chi-square	<0.001^**^
Regurgitation	Chi-square	<0.001^**^
Chest pain	Chi-square	0.42
Weight loss	Chi-square	<0.001^**^
UGIE findings	Chi-square	<0.001^**^
TBS findings	Chi-square	<0.001^**^

**Table 5:** Univariate analysis using Mann-Whitney U test between patients with achalasia versus non-achalasia for continuous variables *: Significant (p value <0.05); **: Highly significant (p value <0.01) ES: Eckadt score

Variables	Median		P value
	Achalasia	Non-achalasia	
Age	40	45	0.021^*^
BMI	20.1	20.5	0.15
ES	7	5	<0.001^**^

Multivariate analysis identified abnormal endoscopic findings (GEJ resistance with dilated esophagus and/or retained food) and delayed esophageal clearance on TBS as independent predictors of achalasia. Specifically, barium retention at both one and five minutes remained a robust diagnostic marker (p=0.0185) and clinical symptoms lost independent predictive value in the multivariate model (Tables 6, 7).​​​​​

**Table 6:** Multivariate logistic regression – statistically significant independent predictors of achalasia (p<0.05) *: Significant (p value <0.05); **: Highly significant (p value <0.01) GEJ: Gastroesophageal junction

Predictor	Coefficient (β)	P value	Interpretation
GEJ resistance only	1.8124	0.006^**^	Strongly associated with achalasia
GEJ resistance with esophageal dilatation and/or food residue	2.3245	0.001^**^	Strongest endoscopic predictor
Barium retention in 1 min >5 cm	1.8806	0.031^*^	Significant radiologic marker
Barium retention in 5 mins >2 cm	3.5012	0.014^*^	Highly predictive of achalasia
Barium retention in 1 min >5 cm and 5 min >2 cm	2.1892	0.018^*^	Independent and robust diagnostic predictor

**Table 7:** Non-significant predictors of achalasia p value >0.05: statistically non-significant

Predictor	P value	Interpretation
Symptoms (dysphagia severity, regurgitation, weight loss, chest pain)	>0.05	Significant in univariate but not multivariate models

## Discussion

This cross-sectional study described the spectrum of EMDs on HRM in a large cohort of patients with non-obstructive dysphagia from eastern India. Our findings reaffirm the role of HRM as an essential diagnostic tool and highlight the predominance of achalasia, particularly Type II, in this population.

In our cohort, achalasia cardia accounted for 53.9% of all EMDs, with Type II being the most prevalent subtype (46.2%). This observation is consistent with data from other Indian studies. Jain et al. reported a 55% prevalence of achalasia, with Type II forming the largest proportion in a multicentric Indian study [1]. Similarly, Bansal et al. observed achalasia in 60% of their study cohort from northern India, and Saha et al. noted Type II as the dominant subtype in patients with dysphagia (n=38) in their eastern India cohort [3,4].

Our findings also align with regional data from Pakistan, where Rehman et al. reported achalasia in 68% of patients undergoing HRM for dysphagia, with Type II being most frequent [7]. Yeh et al. from Taiwan and Goyal et al. from Punjab, India, also reported Type II as the predominant achalasia subtype in their respective cohorts [5,6].

The high proportion of achalasia in our study may reflect referral bias, as patients with prominent dysphagia are more likely to undergo HRM. Nevertheless, the predominance of Type II achalasia may carry prognostic relevance, as this subtype has demonstrated superior response to pneumatic dilation and POEM in published literature [2].

Other significant HRM findings included EGJOO (9.1%), IEM (8.4%), and absent contractility (8.4%). These rates are comparable to those from Goyal et al. and Bansal et al., who reported IEM and EGJOO in approximately 10-15% of dysphagia cases [3,5]. Fragmented peristalsis, a minor disorder, was less frequently identified (1.9%), in keeping with its low clinical yield in non-gastroesophageal reflux disease populations. Goyal and Nagalli observed a 10-20 % chance of concurrent hiatal hernia in achalasia patients; however, our analysis revealed no such cases [10]. Achalasia was associated with longer duration of dysphagia, frequent regurgitation in our study, that align with retrospective study from northern India [11].

Interestingly, 17.9% of patients in our study had normal HRM findings, which may reflect functional dysphagia, early disease, or intermittent motility changes not captured during the test.

Endoscopic and barium tests were non-specific. Our findings underscore that while endoscopy and TBS are useful, they lack sensitivity and specificity in accurately subclassifying EMDs. This reinforces the recommendation that HRM should be the primary diagnostic modality when investigating non-obstructive dysphagia [2,5,12]. Standard diagnostic work-up should include an UGIE, TBS, and manometry followed by additional testing may require if pseudoachalasia suspicion [13]. Abnormal endoscopic and barium findings were independent predictors of achalasia cardia on multivariate analysis, However, UGIE especially done to rule out obstructive lesion [14].

Strengths of this study include its large cohort, standardized HRM diagnostics, and comprehensive correlation with endoscopic and radiologic data. Limitations include its single‑center design and the absence of longitudinal treatment and outcome data, which should be addressed in future prospective studies.

## Conclusions

EMDs in eastern India are predominantly characterized by Type II achalasia, with other significant abnormalities on HRM including EGJ outflow obstruction, IEM, and absent contractility. Endoscopy and TBS can reveal suggestive features of achalasia, such as GEJ resistance and barium retention, but HRM remains indispensable for definitive diagnosis and subtype differentiation. These findings emphasize the need to expand access to HRM and ensure early referral for patients with non-obstructive dysphagia.
